# 

**Published:** 1846-11

**Authors:** 


### CONTENTS

OF NO. V.

### ORIGINAL COMMUNICATIONS.

ESSAYS AND CASES.

Art. I.—An Essay on Scrofula, submitted to the Medical Society of Tennessee, at its annual meeting in May, 1846. By W. L. Sutton, of Georgetown, Kentucky. - - 369

BIBLIOGRAPHICAL NOTICES.

Art. II.—A Practical Treatise on the Diseases of Children. By James Milman Coley, M.D., Member of the Royal College of Physicians in London, &c., &c. - - - 430

Art. III.—A Text-Book on Chemistry for the use of Schools and Colleges. By John William Draper, M.D., Professor of Chemistry in the University of New York, Member of the Philosophical Society, &c., &c. - - - 431

Art. IV.—The Medical Formulary: being a Collection of Prescriptions derived from the Writings and Practice of many of the most eminent Physicians in America and Europe. To which is added an Appendix containing the usual Dietetic Preparations and Antidotes for Poisons. The whole accompanied with a few brief Pharmacentic and Medical Observations. By Benjamin Ellis, M.D., late Professor of Materia Medica and Pharmacy in the Philadelphia College of Pharmacy. Eighth edition, with numerous additions. By Samuel George Morton, M.D., &c., &c. 432

Art. V.—A System of Anatomy for the Use of Students of Medicine. By Caspar Wistar, M.D., late Professor of Anatomy in the University of Pennsylvania. With Notes and Additions. By William E. Horner, M.D., Professor of Anatomy in the University of Pennsylvania. Ninth edition. Entirely remodeled and illustrated by more than two hundred engravings. By J. Pancoast, M.D., Professor of General, Descriptive and Surgical Anatomy in Jefferson Medical College of Philadelphia; &c., &c. - 432

Art. VI.—Human Physiology. With three hundred and sixty-eight illustrations. By Robley Dunglison, M.D., Professor of the Institutes of Medicine, in Jefferson Medical College, Philadelphia; &c., &c. - - - - 433

ART. VII.—A Pocket Atlas of the Descriptive Anatomy of the Human Body. By J. N. MASSE, Pofessor of Anatomy, Paris. Translated from the last Paris edition and edited by GRANVILLE SHARP PATTISON, M.D., Professor of Anatomy in the University of New York; &c., &c. - - 434
ART. VIII.—Scrofula; its Nature, its Prevalence, and the Principles of Treatment. By BENJ. PHILIPS, F.R.S., &c., &c. - - - - - - - - - - - 436
ART. IX.—The Writings of Hippocrates and Galen. Epitomised from the Original Latin Translations. By JOHN REDMAN COXE, M.D., Member of the Batavian Society of Sciences at Haarlem; &c., &c. - - - - - - 436
ART. X.—The United States Dissector, or Lessons in Practical Anatomy. By WM. E. HORNER, M.D., Professor of Anatomy in the University of Pennsylvania. Fourth edition, with numerous illustrations. Edited by Henry H. Smith, M.D., Fellow of the College of Physicians of Philadelphia, &c., &c. - - - - - . - 437
ART. XI.—Braithwaite's Retrospect of Practical Medicine. - 437

SELECTIONS FROM AMERICAN AND FOREIGN JOURNALS.

Heat at Mosul. - - - - - - - - - - 438
Statistics of Consumption. - - - - - - - 438
Treatment of White Swelling. - - - - - - 440
On the Curative Medication of Intermittent Fever. - - 441
On the Employment of Magnetic Electricity in Certain Forms of Paralysis. - - - - - - - - - - - 442
Neuralgia Treated by Colchicum. - - - - - - 445
Case of Poisoning by Opium, in which Electro-Magnetism was Employed. - - - - - - - - - - - 446
A Method of Making the Protoiodide of Iron. - - - - 457
Cases of Diarrhœa, with Emaciation, coming on after Weaning, successfully treated with Creosote. - - - - - 448
Clinical Lectures on Diarrhœa of Infants. - - - - - 450
Tuberculous Abscess of the Lungs opening externally. - 452

ORIGINAL INTELLIGENCE.

Foreign Correspondence. - - - - - - - - - 453
New Medical School. - - - - - - - - - 457
Facts from Correspondents. - - - - - - - 458
General Œdema after the use of Quinine. - - - - - 460

